# Supplementary material for: Ca2+-Daptomycin targets cell wall biosynthesis by forming a tripartite complex with undecaprenyl-coupled intermediates and membrane lipids
Source: Nat Commun. 2020 Mar 19;11:1455. doi: 10.1038/s41467-020-15257-1 (PMC7081307; doi:10.1038/s41467-020-15257-1)
Supplement: Supplementary file 1 — Supplementary Information [file 41467_2020_15257_MOESM1_ESM.pdf]

**Supplementary information**

# $Ca^{2+}$-Daptomycin targets cell wall biosynthesis by forming a tripartite complex with undecaprenyl-coupled intermediates and membrane lipids

**Grein et al.**

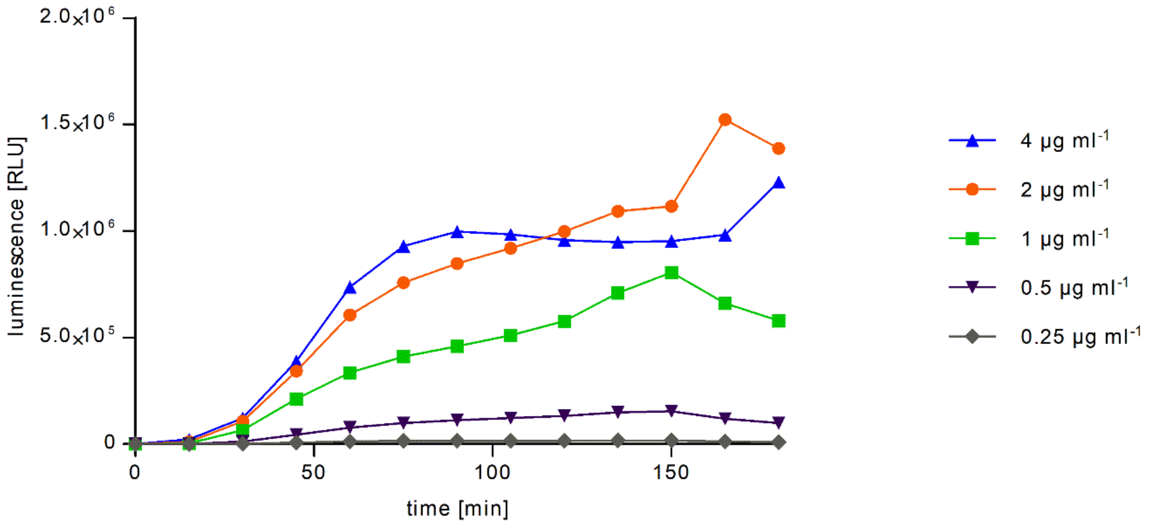


**Supplementary Figure 1: DAP induces the LiaRS-mediated cell envelope stress response in *B. subtilis* in a dose-dependent manner.** Induction of the LiaRS response in *B. subtilis* indicating interference with the lipid II biosynthesis cycle was examined by measuring P*liaI*-*lux* expression over time. Treatment with DAP elicited cell envelope stress at concentrations ranging from 0.5 to 4 µg ml$^{-1}$. Luciferase activity is presented as relative luminescence units (RLU). Representative graph of three independent experiments with comparable results. Source data are provided as a Source Data file.

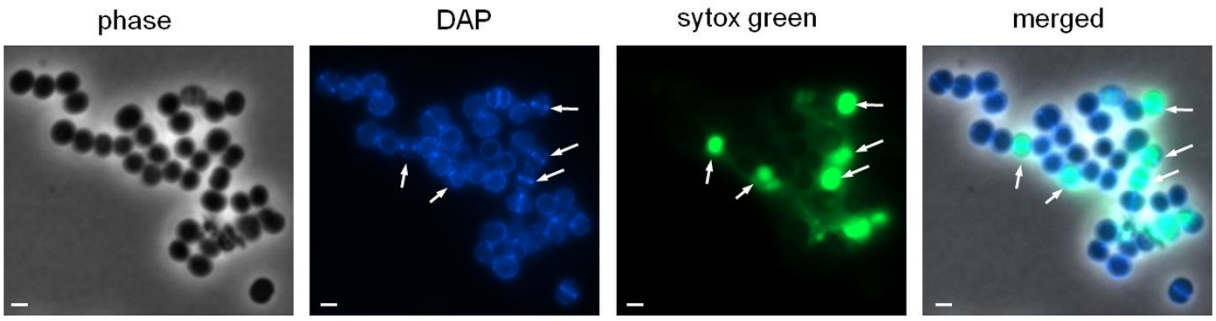


**Supplementary Figure 2: Septal binding of DAP entails killing of *S. aureus* cells.** An *S. aureus* culture was treated with DAP (7 µg ml$^{-1}$) in the presence of $Ca^{2+}$ for 15 min followed by washing of the cells and incubation with sytox green. Cells were washed again and subjected to fluorescence microscopy. Arrows indicate cells with septal DAP localization that are intensively stained by sytox green. Phase, phase contrast. Scale bar 1 µm. Representative images from 3 independent experiments are shown.

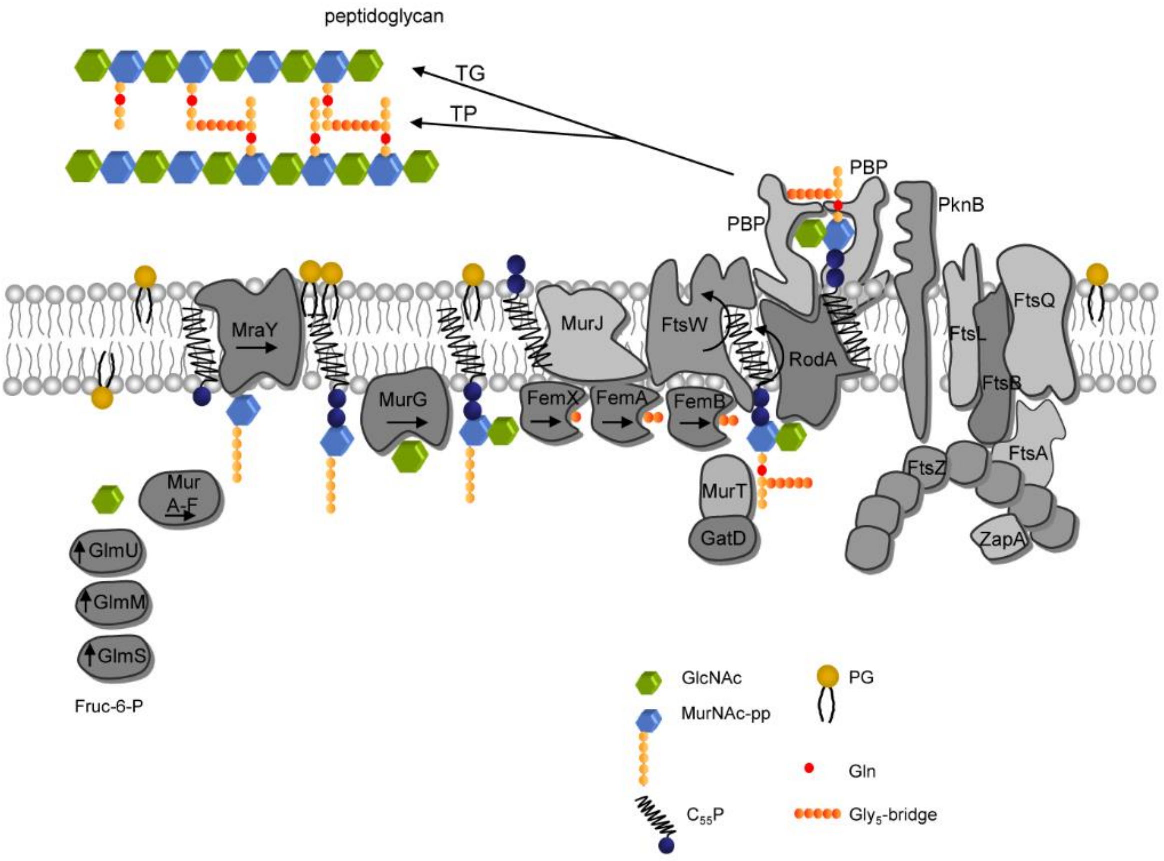


**Supplementary Figure 3: Schematic depiction of peptidoglycan biosynthesis in *S. aureus*.**
The ultimate soluble peptidoglycan precursor UDP-MurNAc-pentapeptide is synthesized in the cytoplasm by the sequential action of MurA to MurF enzymes starting from UDP-GlcNAc provided by GlmS, GlmM and GlmU. The glycosyltransferase MraY transfers the soluble sugar-peptide moiety in the first membrane-associated step to the lipid carrier $C_{55}P$ producing lipid I. MurG subsequently links a GlcNAc moiety to the muramoyl portion of lipid I converting it to lipid II. Lipid II is further species-specifically modified by the addition of five glycine residues catalysed by the peptidyltransferases FemXAB and by amidation of the glutamate residue in position 2 of the stem peptide catalysed by the hetero bi-enzyme complex MurT/GatD. Finally, lipid II is translocated across the cytoplasmic membrane by members of the SEDS or MOP families, and the peptidoglycan monomer is incorporated into the growing peptidoglycan network by transglycosylation and transpeptidation promoted by penicillin binding proteins. GlcNAc, N-acetyl-glucosamine; MurNAc-pp, N-acetyl-muramic acid pentapeptide; $C_{55}P$, undecaprenyl-phosphate; Gly5-bridge, pentaglycine interpeptide bridge; PG, phosphatidylglycerol; Gln, glutamine; TP, transpeptidase; TG, transglycosylase.

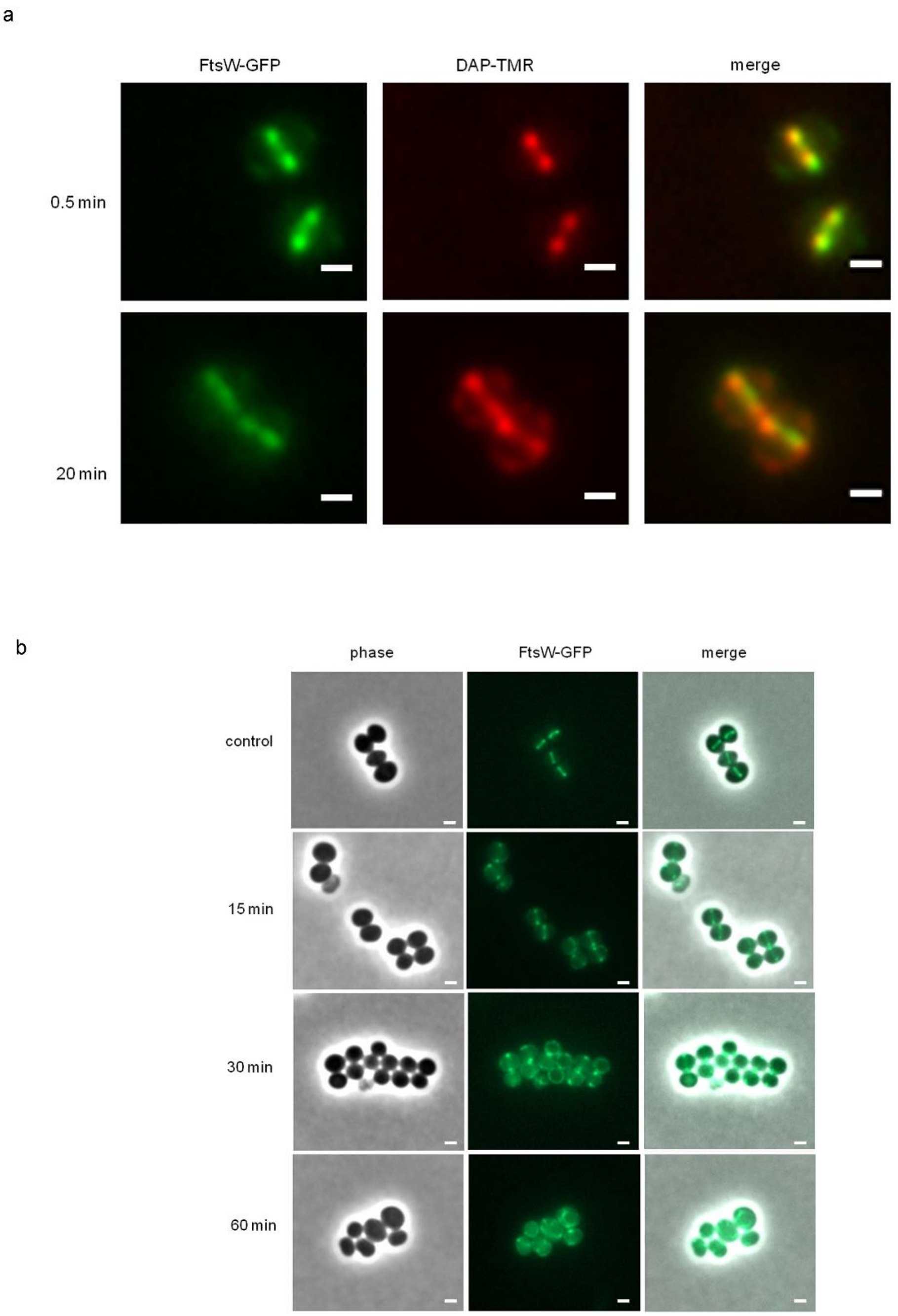


**Supplementary Figure 4: Localization of the putative lipid II flippase FtsW under DAP treatment.** (a) Co-localization of FtsW-GFP and DAP-TMR in *S. aureus* cells during phase I binding. *S. aureus* RN4220 FtsW-GFP was grown to mid-exponential phase ($OD_{600}$ = 0.5) followed by addition of $Ca^{2+}$ and a mixture of labelled and unlabelled DAP (1.6 µg $ml^{-1}$ DAP; 0.16 µg $ml^{-1}$ DAP-TMR). Excess DAP-TMR and non-labelled DAP was removed and cells imaged after 0.5 and 20 min by combining HILO microscopy with dual-color imaging using a dual-emission image splitter. Scale bar 1 µm. Representative images from 2 independent experiments are shown. (b) Sublethal concentrations of DAP lead to delocalization of the putative lipid II flippase FtsW. *S. aureus* cells expressing chromosomally encoded FtsW-GFP were treated with DAP (2 µg $ml^{-1}$) in the presence of $Ca^{2+}$ and the cells subjected to fluorescence microscopy at the indicated time points. Phase, phase contrast. Scale bar 1 µm. Representative images from 3 independent experiments are shown.

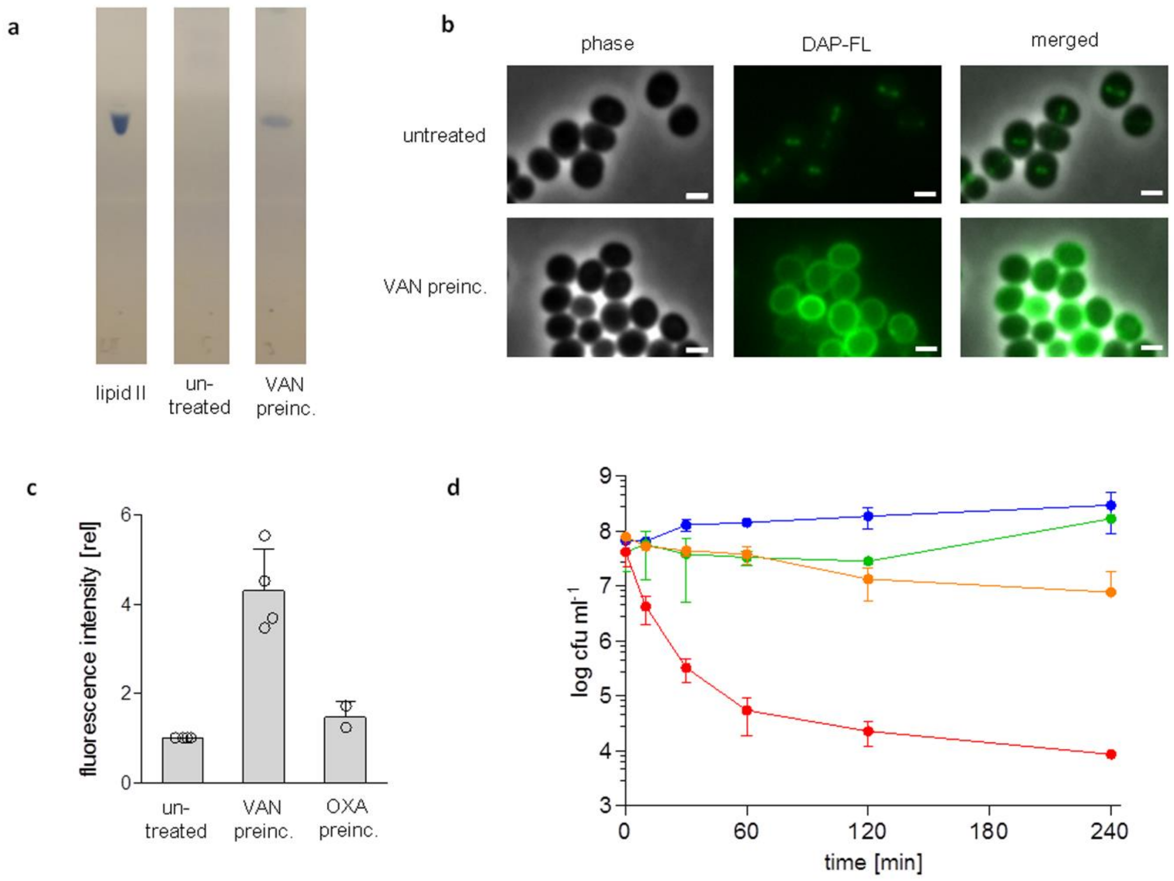


**Supplementary Figure 5: Pre-treatment of *S. aureus* with VAN leads to increased lipid II levels and enhanced binding of DAP-FL. (a)** TLC of lipids extracted from untreated cells (lane 2) or cells treated with VAN (5 µg $ml^{-1}$) (lane 3) for 30 min. Purified lipid II was applied in lane 1. Representative images from 3 independent experiments are shown. **(b)** DAP-FL binding to *S. aureus* cells treated with VAN (5 µg $ml^{-1}$) or oxacillin (5 µg $ml^{-1}$) for 30 min followed by washing the cells twice and incubation with DAP (mixture of DAP-FL and native DAP) for 10 min in the presence of $Ca^{2+}$. Lipid II is expected to be dispersed over the entire cell membrane, as a consequence of the strong accumulation. Scale bar 1 µm. Representative images from 3 independent experiments are shown. **(c)** Quantification of DAP-FL binding measured during the experiment described in (b). Fluorescence intensity of the untreated control cells was set as 1.Values are means from 4 (untreated, Vancomycin preincubated) or two (oxacillin treated) independent experiments. **(d)** Survival of *S. aureus* without treatment (blue line), after treatment with VAN (green line), treated with DAP (5 µg $ml^{-1}$) (yellow line) or treated with DAP (5 µg $ml^{-1}$) after pre-incubation with VAN (red line). Values are means from two independent experiments. Error bars in (c) and (d) represent the SD. Source data are provided as a Source Data file.

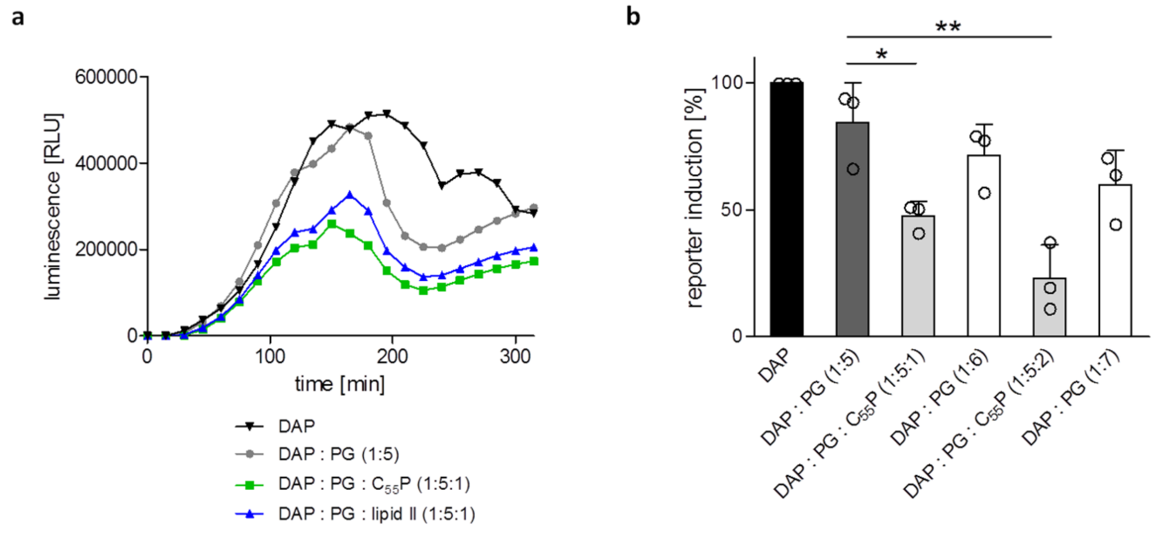


**Supplementary Figure 6: LiaRS induction by DAP is antagonized by pre-incubation with purified cell wall precursors.** (a) *liaI-lux* induction in *B. subtilis* is antagonized when DAP (1 µg $ml^{-1}$) is pre-incubated with $C_{55}P$ or lipid II in presence of PG in molar ratios as indicated. This effect is not observed when DAP is pre-incubated with PG alone. Representative graph of three independent experiments is shown. (b) To exclude unspecific charge effects, the amount of PG was adjusted (white bar) to equalize anionic charges of $C_{55}P$ (light grey bars). Maximal induction relative to the DAP control (black bar / set as 100 %) is shown as mean values  from three independent experiments. Error bars represent the SD.Significance was determined by unpaired Student's t-test with a 95% confidence interval. *p = 0.0184, **p = 0.0065. Source data are provided as a Source Data file.

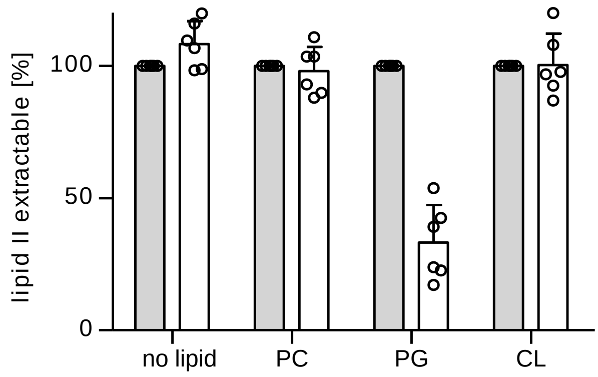


**Supplementary Figure 7: DAP forms an extraction stable complex with lipid II only in the presence of PG.** DAP was incubated with purified lipid II in a molar ratio of 10:1 in the presence of PC, PG or CL or in the absence of lipids (white bars). Controls (grey bars), to which DAP was added immediately prior to extraction, were set as 100%. Reaction mixtures were extracted with BuOH and the upper solvent phase was applied to TLC followed by staining and quantitative analysis of the lipid II band. Data presented are means  from three independent experiments and error bars represent the SD. Source data are provided as a Source Data file.
