# Supplementary material for: Ca2+-Daptomycin targets cell wall biosynthesis by forming a tripartite complex with undecaprenyl-coupled intermediates and membrane lipids
Source: Nat Commun. 2020 Mar 19;11:1455. doi: 10.1038/s41467-020-15257-1 (PMC7081307; doi:10.1038/s41467-020-15257-1)
Supplement: Supplementary file 3 — Description of Additional Supplementary Files [file 41467_2020_15257_MOESM3_ESM.pdf]

Description of Additional Supplementary Files

File Name: Supplementary Movie 1

Description: **3D colocalization of DAP-TMR and FtsW-GFP fusion proteins at the septum.** *S. aureus* cells expressing chromosomally encoded FtsW-GFP were treated with a mixture of DAP-TMR and native DAP, and a z-stack was acquired with a distance of 50 nm between single slices during binding phase I at 0.5 min. For 3D reconstruction, the stacks were processed using the 3D Hybrid Median Filter plugin from ImageJ. Rotations of single FtsW-GFP (green) and DAP-TMR (magenta) as well as merged channels are shown which clearly demonstrate the colocalization of FtsW and DAP to the septal z-ring. Scale bar 1 µm.

File Name: Supplementary Movie 2

Description: **Binding of DAP onto supported lipid bilayers requires both PG and undecaprenyl-coupled cell wall precursors**. Representative movies are shown to demonstrate - with the example of LII - that BDP FL-DAP binding is only slightly increased compared to neutral supported bilayers when they are supplemented with only PG or LII, but largely when both PG and LII are present. The quantitative evaluation of average intensity for all precursors is shown in Figure 5. Ca^{2+}-concentration was 1.25 mM. Movies were recorded with a frame rate of 60 Hz and are slowed down 4 times for display. Scale bar 5 µm.

File Name: Supplementary Movie 3

Description: **DAP does not bind to bilayers pre-incubated with antibiotics specific for single bactoprenyl precursors.** Membranes containing 0.1 mol% PG and 0.1 mol% bactoprenyl lipids ($C_{55}P$, $C_{55}PP$, and lipid II) were pre-incubated with friulimicin, bacitracin, and oritavancin, respectively (antibiotic to bactoprenyl lipids ratio, 100:1) for 5 min. The excess of unbound antibiotics was washed away, and the membranes were incubated with a mixture of BDP FL-DAP and native DAP in the presence of 1.25 mM Ca^{2+}. The membranes were observed and imaged by TIRF microscopy within 2 min after DAP addition. The representative movies displayed show the markedly decreased affinities of BDP FL-DAP for the membranes treated with the corresponding antibiotics, compared to the membranes without the antibiotic treatment, resulting in significant lower fluorescence intensity.
